# Preparation of Hybrid Alginate-Chitosan Aerogel as Potential Carriers for Pulmonary Drug Delivery

**DOI:** 10.3390/polym12102223

**Published:** 2020-09-27

**Authors:** Mohammad Alnaief, Rana M. Obaidat, Mo’tasem M. Alsmadi

**Affiliations:** 1 Department of Pharmaceutical and Chemical Engineering, Faculty of Applied Medical Sciences, German Jordanian University, Amman 11180, Jordan; 2 Department of Pharmaceutical Technology, Faculty of Pharmacy, Jordan University of Science and Technology, Irbid P.O. Box 3030, Jordan; mmalsmadi@just.edu.jo

**Keywords:** drug delivery, hybrid aerogel microparticles, pulmonary delivery, supercritical fluid, acute toxicity

## Abstract

This study aims to prepare hybrid chitosan-alginate aerogel microparticles without using additional ionic crosslinker as a possible pulmonary drug delivery system. The microparticles were prepared using the emulsion gelation method. The effect of the mixing order of the biopolymer within the emulsion and the surfactant used on final particle properties were investigated. Physicochemical characterizations were performed to evaluate particle size, density, morphology, surface area, surface charge, and the crystallinity of the preparation. The developed preparation was evaluated for its acute toxicity in adult male Sprague-Dawley rats. Measurements of zeta potential suggest that the surface charge depends mainly on the surfactant type while the order of biopolymer mixing has less impact on the surface charge. Chitosan amphiphilic properties changed the hydrophilic-lipophilic balance (HLB) of the emulsifying agents. The specific surface area of the prepared microparticles was in the range of (29.36–86.20) m^2^/g with a mesoporous pore size of (12.48–13.38) nm and pore volume of (0.09–0.29) cm^3^/g. The calculated aerodynamic diameter of the prepared particles was in the range of (0.17–2.29 µm). Toxicity studies showed that alginate-chitosan carrier developed herein caused mild lung inflammation with some renal and hepatic toxicities.

## 1. Introduction

Pulmonary drug delivery is an attractive route for drugs used in both local and systemic treatment [1,2,3,4]. Many challenges exist in the manufacturing stages of inhalations for pulmonary drug delivery systems [5,6,7,8,9]. Nevertheless, the proven efficiency of this route for many drugs pushes toward finding practical solutions. Both local medications such as asthmatic drugs, and systemic drugs like insulin [10], hormones [11,12], and anticancer drugs [13] proved to have higher efficiency in the pulmonary route in comparison with other routes of administration.

Pulmonary drug particles include liposomes, micelles, and polymeric drug particles [14]. Polysaccharides and other natural polymers including gelatin, chitosan, and alginate have attracted researchers for their potential application in the biomedical field due to their biocompatibility, biodegradability, and tailored chemical and structure [15,16,17,18].

Due to their high loading capacity, polysaccharides based materials were suggested as a drug particle for many delivery applications [19,20,21,22]. Moreover, hybrid polymers find their way as drug particles [23,24,25]. Blends of chitosan-alginate polymers showed promising uses in controlled release preparations [26,27] and preparations of micro and nanoparticles for drug delivery especially for protein delivery application [28,29,30,31]. These studies were based on the addition of cationic chitosan on anionic alginate polymer in the presence of calcium chloride as a cationic electrolytic cross-linker. However, using calcium chloride in pulmonary particles was opposed by many researchers who discussed the stimulation of inflammatory response from the presence of calcium ions in alginate hydrogels [32], which is undesirable in the lungs. On the other hand, biodegradability can be affected by the presence of such a relatively stable cross-linker. Penhasi shows that the degree of degradation of pectin film can be directly associated with the concentration of CaCl_2_ solution for colon targeted delivery [33].

Due to the ionic nature of alginate and the cationic nature of chitosan, it is possible to generate a 3-D network structure between them based on the electrostatic interaction between the functional groups of the biopolymers. The characteristics of the resultant hybrid polyelectrolyte depend on many factors such as polymer concentration, charge density, molecular weight, ionic strength, pH of the mixture, reaction temperature, and mixing ratio and procedure [34,35]. Recently, increasing applications are proposed for alginate/chitosan polyelectrolyte including food engineering [36,37], a free-standing membrane for drug delivery, wound dressing application [38,39], and drug delivery [40].

This study aims to prepare nanoporous dry particles based on chitosan-alginate polyelectrolyte as a potential carrier for pulmonary drug delivery using the emulsion-gelation technique. Two surfactants were used to produce w/o emulsion. The supercritical carbon dioxide extraction was used to extract the solvent within the gel network of the particles. To the best of our knowledge, no reports are available in the literature for the production of hybrid alginate chitosan nanoporous particles without additional cationic crosslinker or their potential for a pulmonary delivery application using supercritical fluid technology.

## 2. Materials and Methods

### 2.1. Materials 

Chitosan, alginate, was provided by AZ Chem for chemicals, China. Water (HPLC grade), HPLC column C18, Span 85, and span 80 were purchased from Sigma-Aldrich, St. Louis, MO, USA. Potassium bromide (IR spectroscopy grade, Dieuze, France), Hydrochloric acid (37% *w*/*w*) was supplied by Biosolve, France. Absolute ethanol was provided by Solvochem, Holland. Carbon dioxide (CO_2_) with a purity of 99.995 was provided by the Jordanian Gas Co., Amman, Jordan. All chemicals were used as supplied without further modification except for chitosan. Chitosan oligomers (13 kDa) as water-soluble hydrochloride form were prepared from raw chitosan (250 kDa) as reported by Obaidat et al. [41], and molecular weight determination was performed using the Mark-Houwink equation [42].

### 2.2. Preparation of Composite (Alginate-Chitosan) Nanoporous Particles

Aerogel micro-particles were prepared using the emulsifying-gelation method. Briefly, a certain amount of paraffin oil containing 4% (*w*/*v*) surfactant (span 80 or Span 85) was mixed using a homogenizer at 4000 rpm for two minutes. Thereafter, a 2% aqueous chitosan solution was added to the oil phase and mixed for 15 min. Finally, an equal amount of 1% aqueous alginate solution was added to the emulsion and mixed further for another 15 min. The effect of the biopolymer addition sequence was investigated by adding the alginate first followed by chitosan and the simultaneous addition of alginate-chitosan to the oil phase. The produced hybrid particles were then separated from the oil phase using centrifugation at 3000 rpm for 15 min. After that, a successive solvent exchange process was implemented to replace pore liquid with ethanol as stated in previous work [43,44]. Wet alcogel particles were then placed into a 500 mL cylindrical stainless-steel vessel. CO_2_ was pumped into the vessel at a constant flow rate (100–120 g/min), the system pressure and temperature were maintained about 100 bar and 40 °C, respectively. The loaded sc.CO_2_ was directed to another cylindrical stainless-steel vessel, where the solvent was separated from CO_2_. The pressure and temperature of the separator were controlled at 60 bars and 40 °C respectively to allow phase separation of the CO_2_-solvent mixture. Solvent-lean CO_2_ was then recycled to the extraction autoclave [45]. 

Six samples were prepared in this work as listed in Table 1. Samples were prepared with two different surfactants (span 85, span 80) at 4% of the oil phase concentration. The order of addition of the polymer to the oil phase was also evaluated.

### 2.3. Physicochemical Characterizations of the Prepared Particles

#### 2.3.1. Measurement of Particle Size 

The particle size distribution of the prepared particles was determined using a laser particle size analyzer. The average particle size was expressed as the mean volume (MV) in units of a micrometer. Each measurement was repeated three times.

#### 2.3.2. Zeta Potential

A small amount of each formulation dispersed in 5 mL of distilled water then the zeta potential and particle size were measured using a Malvern (ZEN 3600) instrument at 20 °C. Each measurement was repeated three times.

#### 2.3.3. Surface Area and Porosity Analysis

The samples were degassed at 343 K for 24 h before the analysis. low-temperature nitrogen adsorption-desorption analysis (Quantachrome NOVA 2000, USA) was used to determine the specific surface area and the pore volume of the investigated sample following Brunauer–Emmet–Teller (BET) and Barrett–Joyner–Halenda (BJH) methods, respectively. 

#### 2.3.4. Tapped Density Measurement

The tapped densities of the prepared particles were determined using a powder integrative characterization apparatus. A known weight of each sample was placed in a 10 mL cylinder and tapped until a constant volume is reached. The volume was recorded and used to calculate the tapped density according to the following equation:

Tapped density = weight of the sample (g)/Volume of the sample in the cylinder after tapping (cm^3^) (1)

Each measurement was repeated three times.

#### 2.3.5. True Skeleton Density Measurement

The real density of each of the prepared particles was determined using the Helium Ultra pycnometer. Each measurement was repeated five times. 

#### 2.3.6. Aerodynamic Diameter (DA)

The aerodynamic diameter was calculated based on a theoretical approach that was also employed by [46] based on the equation:

(2) $$d_{a}=\;d_{p}\sqrt{\frac{\rho_{e}}{\lambda \rho_{s}}}\;$$

where: *d_a_* is the aerodynamic diameter (µm), *d_p_* is the particle diameter (µm), *ρ_e_* is the effective particle density(g/cm^3^), *ρ_s_* is 1 g/cm^3^, and *λ* is the dynamic shape factor of the particle [46,47,48,49]. The equation suggests that the aerodynamic diameter is a shape-dependent, which in turn depends on the drag forces acting on the moving particles. Davies had intensively reviewed the effect of different particle shapes on its aerodynamic diameters. For pollen shape with a rough surface, like those produced in this study, the dynamic shape factor is larger than unity (for spherical particle *λ* = 1), and 1.2 was estimated for this shape [47].

#### 2.3.7. Fourier Transform Infrared Spectroscopy (FTIR)

FTIR study was also carried out using IRAffinity-1 Spectrophotometer (Shimadzu, Kyoto, Japan) with KBr as a reference. 30 mg of each sample was physically mixed with 270 mg KBr in a mortar with a pestle, and then measured by the instrument. The IR spectrum was acquired in the range from 4000–450 cm^−1^. 

#### 2.3.8. Powder X-Ray Diffraction (PXRD)

Powder X-ray diffraction patterns were carried out using Ultima IV X-ray diffractometer (Rigaku, Japan) with cobalt radiation (CuKα) at a voltage of 40 kV and a current of 30 mA at room temperature with diffraction angles from 0° to 60° of 2θ. The step scan mode was used with a step size of 0.02°.

#### 2.3.9. Differential Scanning Calorimetry (DSC)

Differential scanning calorimetry (DSC) measurements were carried out using DSC 204 (Netzch, Germany). Indium was used to calibrate temperature and energy scale. An accurately weighed sample was placed in a sealed aluminum pan, then it was heated up to 200 °C under constant nitrogen flow at a rate of (30 mL/min). An empty sealed aluminum pan was used as a reference.

#### 2.3.10. Thermogravimetric Analysis (TGA)

Thermogravimetric TGA analysis was carried out using the TG 209 F1 Iris (Netzch, Selb, Germany). An accurately weighed sample was heated from 30 to 300 °C at a rate of 10 °C/min under a nitrogen purge flow of 50 mL/min. The percentage of the mass loss was calculated based on the mass of the original sample.

#### 2.3.11. Scanning Electron Microscopy (SEM)

The surface morphology of the samples was obtained using Quanta FEG 450, SEM (FEI, felmi-zfe, Graz, Austria). Before performing SEM analysis, the samples were placed on stubs and coated with platinum under a vacuum atmosphere using Q150R Rotary-Pumped Sputter Coater/Carbon Coater (Quorum Technologies, Laughton, East Sussex, UK).

#### 2.3.12. Yield 

To determine the yield, the amount of product obtained from each preparation was weighed, and the yield percent was calculated by using the following equation:

Yield % = (Weight of prepared sample(g)/Total weight of the polymers used for the preparation(g)) × 100%(3)

### 2.4. Statistics

Each experiment was conducted at least 3 times. The data were presented as mean ± standard deviation.

### 2.5. In Vivo Toxicity Studies

Being evaluated for their physicochemical characteristics, the developed carrier was then evaluated for its acute toxicity in adult male Sprague- Dawley rats (180 g). 35 mg/kg of the carrier powder was reconstituted in 0.2 mL normal saline and slowly (over 20 s) administered directly into the trachea to semi-anesthetized (by ether) using 1 mL syringe. All of the procedures applied to rats were approved by the Animal Care and Use Committee (ACUC) at Jordan University of Science and Technology that follows the international IACUC rules. Twenty-four rats were randomly divided into 4 equally-sized groups: 

Group 1 received intratracheal saline and was used as a control, group 2 received alginate raw powder, group 3 received chitosan raw powder, and group 4 received alginate-chitosan developed carrier,

Rats were clinically observed before and after carrier administration and they had free access to water and food during the experiments. After 24 h of receiving the treatment, the rats were weighed and necropsy was achieved by ether overdose. The lungs, liver, and kidneys were collected and stored in 10% formalin. Tissue slides were examined by light microscopy after staining with hematoxylin and eosin.

## 3. Results

The polyelectrolytes composites microparticles were prepared with the yield values exceeding 70% for all prepared samples using span 85, while the yield value of span 80 preparations did not exceed 40%. 

### 3.1. Structural Properties

Table 2 shows the textural properties of the prepared samples in this study. Samples 1, 2, and 3 were prepared using Span 85 as a surfactant, while samples 4, 5, and 6 were prepared using Span 80 as a surfactant. The average particle size for the preparation prepared using Span 85 were ranging from 0.433 ± 0.091 µm for sample 1 to 4.170 ± 0.480 µm for sample 2. On the other hand, Nano-sized particles were obtained using Span 80 ranging from 70± 43 nm for sample 4 to 84 ± 62 nm for sample 5. Particle size results can show high polydispersity for the samples prepared using Span 80 compared to Span 85. Samples prepared using span 85 tend to have positive Zeta potential values ranging from 35.4 ± 5.37 mV for sample 2 to 45.3 ± 3.44 mV for sample 1. On the other hand, samples 4, 5, and 6 have much lower zeta potential values ranging from −2.15 ± 3.70 to −5.98 ± 5.37 mV. Samples prepared using Span 85 have a higher specific surface area compared to those prepared using Span 80. The specific surface area for the prepared samples was ranging from about 29 m^2^/g for sample 5 up to 86 m^2^/g for sample 1. The sample 3 surface area was below the detection limit of the nitrogen sorption device. All prepared samples, excluding sample 3, exhibit mesoporous diameter in the range of 12.48–13.38 nm. 

**Figure 1:**
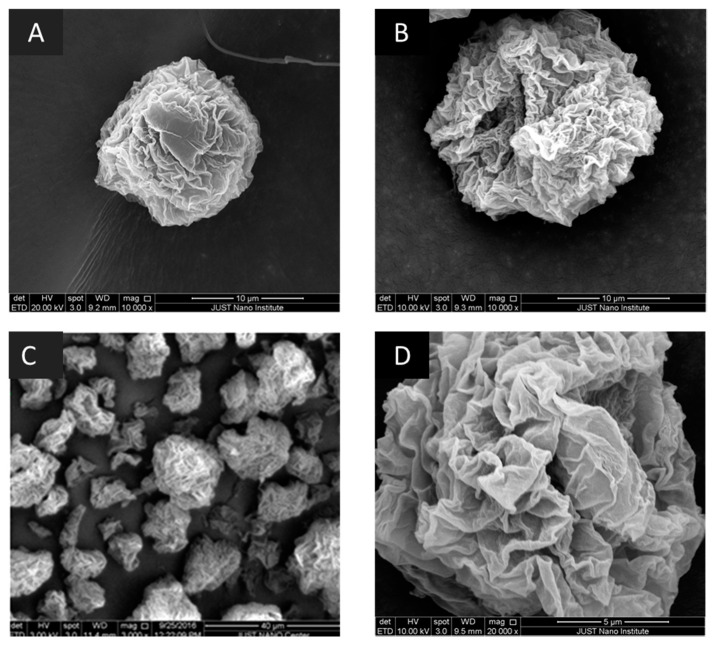
Scanning electron microscopy (SEM): (**A**) images for S1 at 10,000×; (**B**) image for S2 at 10,000×; (**C**) Sample S4 at 3000×; (**D**) Sample S4 at 20,000×. Selected samples are presented with acceptable calculated aerodynamic diameter.

Figure 1 shows model SEM images at 10,000× magnification for the prepared particles for samples 1 and 2. The roughness of the surface of the prepared sample was clear in SEM images. The same roughness and morphology were obtained for samples prepared using Span 80, where the three samples (4, 5, and 6) had similar images. This can be observed in the model SEM images for one of the samples prepared using Span 80 at magnifications 3000, and 12,000×. Moreover, the SEM images show that some of the particles can have a particle size larger than 5 µm.

### 3.2. Physicochemical Characterizations of the Prepared Particles

#### 3.2.1. FTIR Analysis

**Figure 2:**
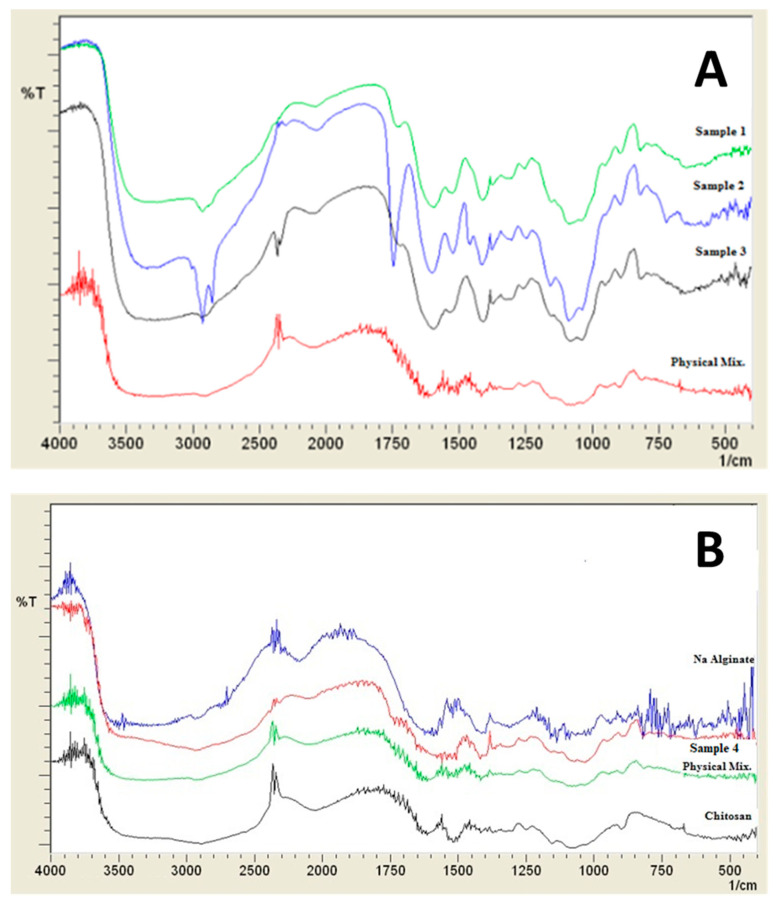
Infra-red spectra of prepared samples (**A**) Samples 1, 2, 3, and their corresponding physical mixture, (**B**) Na alginate, chitosan, sample 4, and its corresponding physical mixture.

Figure 2 shows the FTIR scan for the prepared samples compared to the corresponding physical mixture. The typical bands of chitosan and alginate appeared in the FTIR spectrum of the physical mixture [50]. Sample 1 and 2 show clear characteristic peaks for chitosan without major changes due to the hybridization with alginate which emphasizes maintaining the chemical entity of the polymers. While some shifts were observed in samples for the peaks related to amide I and amide II peaks at 1650 cm^−1^ and 1600 cm^−1^, respectively, with the appearance of a new peak around 1160 cm^−1^. This peak is characteristics of saccharide structure and can be related to the anti-symmetric stretching of the C-O-C bridge [50]. For samples 4, 5, and 6, the peak at 2876 cm^−1^ is caused due to OH stretching of chitosan, while the peak at 1655 cm^−1^ is due to an absorption band of C=O. Moreover, the symmetric stretching of COO^−^ was shifted to 1415 cm^−1^. A shift was also observed for 1599 cm^−1^ peak, which is responsible for NH bending vibrations to 1559 cm^−1^. Generation of a carboxyl group peak at 1750 cm^−1^ in all prepared samples can be related to symmetric and asymmetric stretching of –COO– groups [50].

#### 3.2.2. DSC Analysis

**Figure 3:**
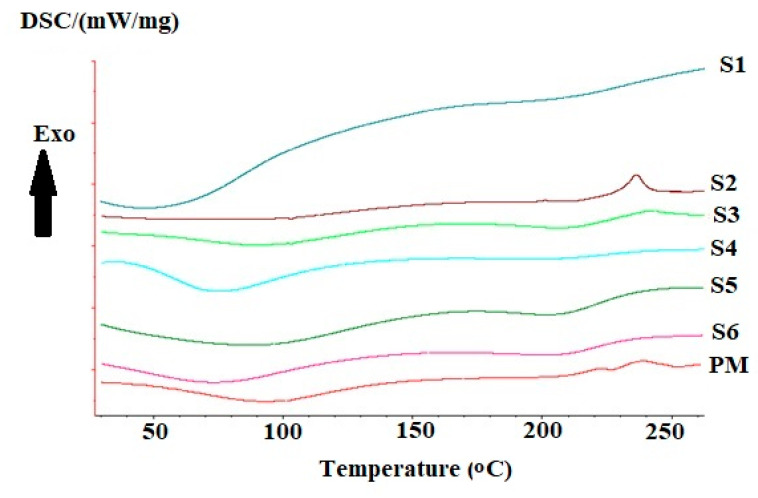
Differential scanning calorimetry thermograms of prepared samples 1, 2, 3, 4, 5, 6, and the corresponding physical mixture.

Thermal analysis can help in investigating the interactions between polymers. The DSC thermograms are presented in Figure 3. The physical mixture thermogram shows an initial shallow endothermic peak that started below 50 °C and extended above 100 °C, this peak can be related to water loss associated with the hydrophilic groups of the two polymers. Another exothermic peak appeared around 230 °C, and 250 °C. These peaks can be related to the decomposition of chitosan, and alginate, respectively [51]. Some changes occurred in the thermograms of chitosan-alginate samples. These include shifts in the present endothermic peaks. The first peak was shallower in the first three samples (1, 2, 3) compared to other samples.

Samples prepared using Span 85 showed a large increase in the area of the endothermic peak in sample 1 with the appearance of two small peaks at 100 °C and 130 °C. While sample 2 shows a clear difference from other samples with a lack of endothermic peak that is related to chitosan or alginate compared to the physical mixture. On the other hand, sample 3 showed the appearance of the endothermic peak without any difference from that of the physical mixture. For samples prepared using span 80, both samples 4 and 5 showed a clear appearance of alginate related peak without significant difference between peaks of sample 6 and the corresponding physical mixture. 

#### 3.2.3. TGA Analysis

**Figure 4:**
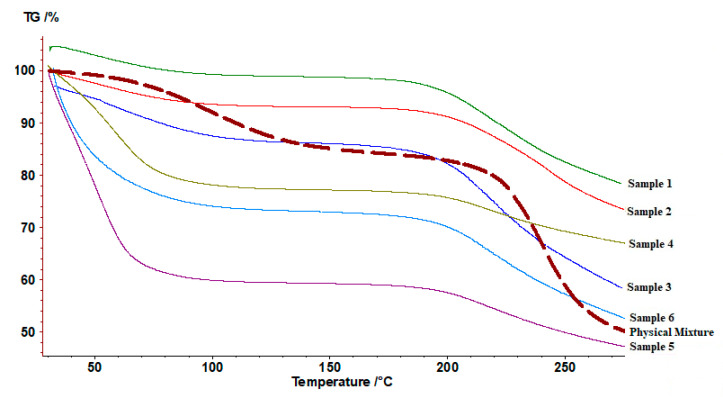
Thermogravimetric thermograms of prepared samples 1, 2, 3, 4, 5, 6, and the corresponding physical mixture.

TGA thermograms (Figure 4) exhibited two stages of weight loss. The first one can be related to water content inside the sample. The presence of moisture content is expected for chitosan, and alginate hydrophilic polymers. A clear difference in the water content of the first three samples (1, 2, 3) and all other samples including a physical mixture. The water loss in the first three samples has an average value of 9.4 ± 2.4%, compared to 44.3 ± 10.6% in the other three samples (4, 5, 6). While the water content for the chitosan-alginate physical mixture equals 15.2 ±1.6%. On the other hand, the second stage can be related to polymeric degradation. All samples exhibited slightly lower degradation temperatures starting from 200 °C compared to 230 °C for the physical mixture. 

#### 3.2.4. PXRD Patterns

**Figure 5:**
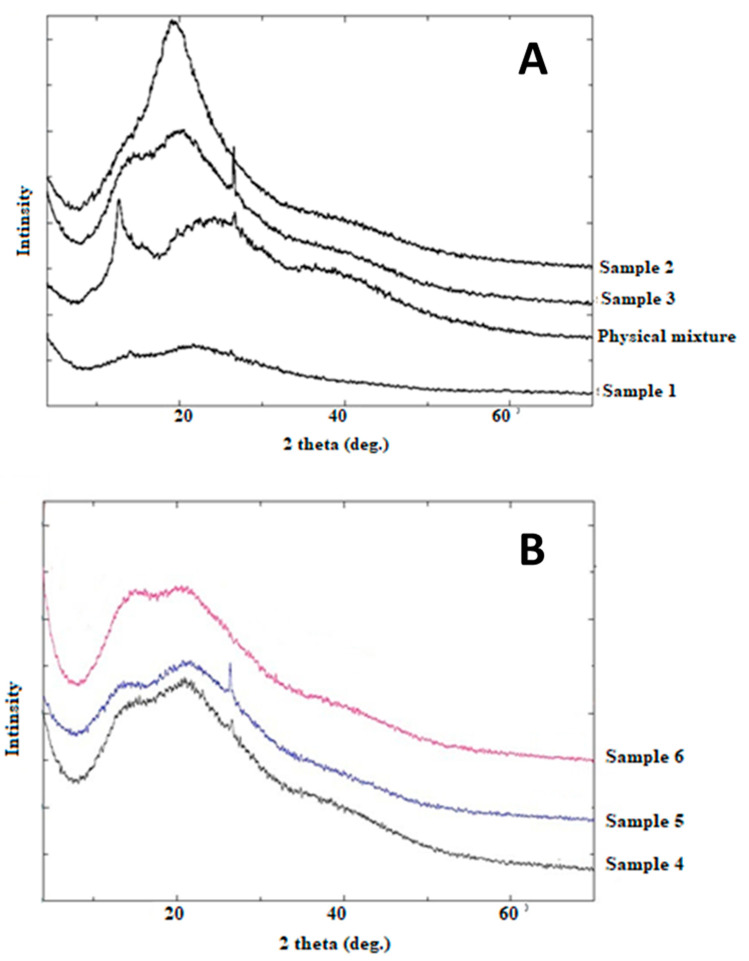
Powder X-ray diffractions of (**A**) Samples 1, 2, 3, and their corresponding physical mixture, (**B**) Samples 4, 5, and 6.

Both samples 1 and 2 showed different Powder X-Ray Diffraction (PXRD) patterns (Figure 5) from the physical mixture. The shallow peak in sample 1 is indicating a change into an amorphous structure. On the other hand, the presence of a main shallow peak at (2θ = 20°) can be used as indicative of a specific arrangement of the two polymers. Generally, the other samples show a pattern similar to the physical mixture with minor differences that can indicate a partial change in the sample.

### 3.3. In Vivo Toxicity Studies

**Figure 6:**
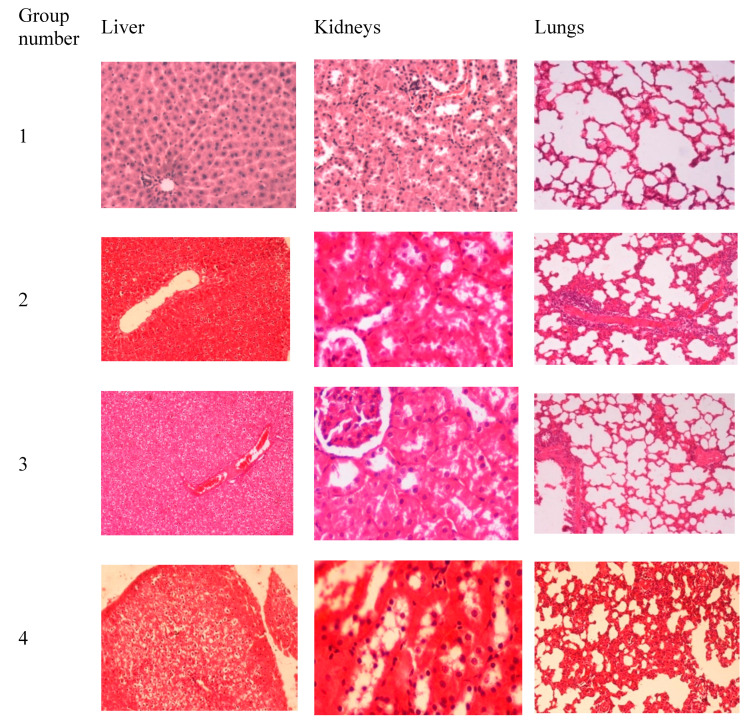
The results of histopathological examination of the liver, lungs, and kidneys harvested from rats receiving intratracheal: saline (group **1**), alginate raw powder (group **2**), chitosan raw powder (group **3**), and alginate-chitosan carrier (group **4**). All of the tissue slides were magnified at 200×.

Control group rats (group 1) showed normal tissue sections (Figure 6). The lungs showed mild thickening in the alveolar septa and the liver showed vacuolar degeneration in all of the rats receiving intratracheal alginate (group 2), chitosan (group 3), and alginate-chitosan carrier (group 4). On the other hand, while group 2 and group 3 had normal kidneys, group 4 showed tubular degeneration and necrosis with some proteinous materials.

## 4. Discussion

### 4.1. Structural Properties

Alginate solution as well as chitosan solution were subject to the used method and tested if they can yield a stable gel to be dried later using supercritical carbon dioxide. Both preparations failed to gel and remain in the liquid form. However, all samples in this study result in the production of porous particles except for sample 3 (Table 2). This is an indication of the polyelectrolyte complexation between alginate and chitosan that results in a stable 3D network that can be further processed using supercritical carbon dioxide extraction to generate the nanoporous dried particles. Delaney and Fredrickson show that mixing two oppositely charged polyelectrolyte in a polar phase can result in polyelectrolyte complexes that solidify upon formation. These kind of polyelectrolyte complexes are difficult to understand since they are highly dependent on the processing pathway [52]. Samples 1 and 2 showed clear differences in PXRD, high positive zeta potential values, and highest surface area compared to other samples indicating efficient complexation. The deviation of zeta potential values between samples suggests a different arrangement between the two chains of the polymers. Chitosan is a cationic polymer, so having positive zeta potential value is indicative of high contribution to chitosan at the surface. On the other hand, negative zeta potential values are indicative of high contribution to alginate anionic polymer. High zeta potential values suggest that particles can stay physically stable with less aggregation. Both samples 1 and 2 show high zeta potential values.

Particle properties were affected by surfactant type and order of polymeric addition. Differences observed in the particle size, surface charge, pore size, pore-volume, and crystallinity of the prepared samples.

Further calculation of aerodynamic parameters according to Hassan et al. 2009, proved good results with values lower than 5 µm ranging from 0.17 to 2.29 µm, yet the values in this study are calculated and further actual cascade impactor experiments and in-vivo deposition tests are required [46]. Vanbever et al. reported that for porous particles the particle geometric diameter can be larger than 5 µm for inhalation delivery. Such porous particle have the advantage of less aggregation and can be deaggregated more easily than smaller particles [53]. Larger geometric diameters are possible since the aerodynamic diameter is directly proportional to the square root of the effective particle density.

Looking at the textural properties of the prepared samples (particles size, surface area and pore volume) it is clear that the samples prepared using lower hydrophilic-lipophilic balance (HLB) value (span 85) and by adding chitosan to alginate shows the best properties to in terms of yield, optimum particle size, aerodynamic parameter, and high zeta potential values. Further investigation is still required to bring these two samples (samples 1 and 2) forward as a particle for inhalation delivery especially that some other critical parameters can play an important role in particle deposition. These include gelling and swelling of the particles in the presence of relative humidity, mucoadhesion, and zeta potential.

### 4.2. Effect of Changing the Surfactant Type on Particle Properties

Span 80 and span 85 were investigated as surfactants with low HLB values of 4.3 and 1.8, respectively, to prepare w/o emulsion. The surfactant type appeared to have an important role in the properties of the prepared particle. The required hydrophilic-lipophilic balance (RHLB) equals 4.3 and span 80 was supposed to be the best emulsifying agent to prepare a successful emulsion. Yet span 80 failed to produce particles with optimum properties of particle size and surface charge. Span 80 resulted in lower zeta values with the presence of aggregates in the prepared samples. On the other hand, researchers decided to try span 85 with lower HLB value, the specific amount of the surfactant was chosen according to preliminary experiments. A successful emulsion was produced yielding the best particle properties with the highest positive zeta values. Consequently, this reduced the aggregation in the prepared samples. Interestingly, these results confirm the emulsifying effect of chitosan. This suggests that chitosan affected the total RHLB of the emulsion by combined effect between chitosan and the surfactant. The positive charge for samples prepared using span 85 suggested chitosan alignment on the outer layer of the particles due to the presence of chitosan at the oil-water interface. Some previous studies discussed such emulsifying effect of water-soluble chitosan and alignment of the molecule at the interface [54,55]. This interesting finding emphasizes the importance of consideration of the emulsifying property of water-soluble chitosan when enrolled in the preparation of emulsions. Chitosan is expected to change the HLB of the emulsifying agents. In this study, hydrophilic chitosan has a higher HLB value that is combined with 1.8 value for span 85 resulted in the achievement of the RHLB value of the emulsion (4.3) suggesting alignment of chitosan molecules near the span 85 molecules at the (*w*/*o*) interface. Further investigation will be required to define HLB values of chitosan oligomers according to molecular weight and degree of acetylation. Additionally, other physicochemical characterizations revealed changes in the particle characteristics in the samples prepared using different surfactants.

### 4.3. Effect of the Order of Addition of the Two Polymers on Particle Properties

The order of addition played a significant role in particle properties. Higher values of zeta potential, lower particle size, and higher surface area values were achieved when chitosan was added to alginate rather than the opposite. The uniformity in the particle size can be seen in samples 1, 4, 5, and 6 with a standard deviation lower than 0.09. Further investigation should be performed to study the effect of rheological properties of the selected polymeric concentration on these properties. 

### 4.4. Physicochemical Characterizations of Prepared Polyelectrolyte Composite Particles

FTIR analysis was used to prove the polyelectrolytic complexation between chitosan and alginate. Some differences were obtained between samples prepared using span 85 and span 80. Similar shifts in carboxyl groups to 1415 cm^−1^ were reported by Li et al. [30]. Variability in the intensity of the peak at 1750 cm^−1^ (COO^−^) was observed upon changing the order of addition of the polymer. Still, FTIR alone might not be enough to illustrate the polyelectrolytic complexation. Moreover, differences were reported in PXRD analysis especially for samples 1 and 3, which also suggested differences in the final prepared composite particles. This also suggests efficient polyelectrolytic complexation. The interesting discrepancy between the PXRD patterns of the prepared samples confirms that each preparation method resulted in a different type of arrangement of the polymers.

In comparison, the results in SEM analysis showed differences in the external morphology of the sample. These differences are very critical in pulmonary drug delivery particles. Yet, further in-vivo pulmonary deposition tests can clarify the morphology effect.

### 4.5. In Vivo Toxicity Studies

The safety of the pulmonary administration of carriers can be enhanced by being biodegradable [2]. Intratracheal administration of alginate powder, chitosan powder, and alginate-chitosan composite hydrogel resulted in mild lung congestion. Previous reports showed that the intratracheal administration of polymers caused mild inflammation of the lungs [56]. Moreover, intratracheal administration of alginate-chitosan carrier caused some haptic toxicity. This contradicts previous reports where oral administration of the same carrier in Guinea pigs did not cause any hepatic abnormalities [57]. This could be attributed to differences in studied species (rats versus Guinea pigs), route of administration (intratracheal versus oral), or the alginate-chitosan carrier preparation technique (supercritical fluid technology versus chemical crosslinking using calcium chloride). Overall, the novel carrier produced in the current work was tolerable by the studied rats at the dose received when administered for one month [45]. Both chitosan and alginate are known to be biocompatible polymers [58,59]. Biocompatibility is defined as “Ability to be in contact with a living system without producing an adverse effect” [60]. Biodegradable, is “Qualifier for a substance or device that undergoes biodegradation” [60]. Biodegradation is “Degradation of a polymeric item due to cell-mediated phenomena”, that is not merely due to contact with tissue water which is known as hydrolysis in this case [60]. The developed carrier (S1) was used to deliver cisplatin to the lungs for lung cancer treatment. In that study, it was shown that the carrier allowed for sustained release of cisplatin and reduced the cisplatin-induced lung toxicity, mortality rate, and weight loss in rats treated with cisplatin loaded carrier as compared to rats treated with free cisplatin [45]. 

## 5. Conclusions

Chitosan-alginate nanoporous particles were prepared without the addition of any cross-linker. In preparation of chitosan-alginate composite polymers, two processing parameters affected the properties of the prepared particles. These include the order of polymeric addition during the gelling process, as well as the type of surfactant used for the emulsification process. The final properties of the prepared particles that were affected by the studied parameters were: porosity, specific surface area, shape, crystalline state, surface charge, and zeta potential values. High positive zeta potential values were obtained for samples prepared using span 85, while negative zeta values and smaller particle sizes were reported for all span 80 samples. Negative zeta potential values suggest that alginate is surrounding chitosan oligomers, while positive values may indicate that chitosan surrounds the core of alginate. Chitosan amphiphilic properties changed the HLB of the emulsifying agents. Prepared particles had variability in specific surface area values that reached 86.2 m^2^/g. Particle sizes were in the range from 70 nm to 4.17 µm. Physicochemical characterizations proved different arrangements and crystalline states depending on the order of polymeric addition. These variables affected the value of the calculated aerodynamic diameter which ranged from 0.17 to 2.29 µm. In conclusion, chitosan-alginate hybrid composites can be optimized for pulmonary drug delivery systems. The preparations are ready for further biodegradability tests, cascade impactor, and drug loading studies.

## Figures and Tables

**Table 1:** Samples IDs and preparation conditions used in this work.

Samples	Surfactant Type	Order of Polymer Addition to the Oil Phase	Yield (%)
S1	Span 85	Chitosan added to Alginate	73 ± 5
S2	Span 85	Alginate added to Chitosan	78 ± 6
S3	Span 85	Both polymers are added simultaneously	72 ± 8
S4	Span 80	Chitosan added to Alginate	44 ± 3
S5	Span 80	Alginate added to Chitosan	39 ± 5
S6	Span 80	Both polymers are added simultaneously	59 ± 10

**Table 2:** Physical characterization of the prepared microparticles.

Sample	Size (µm)	Zeta Potential (mV)	Specific Surface Area (m^2^/g)	Porosity (cm^3^/g)	Pore Diameter (nm)	Bulk Density (g/ cm^3^)	Tapped Density (g/ cm^3^)	Calculated Aerodynamic Diameter (µm)
Span 85								
S1	0.433 ± 0.091	45.3 ± 3.44	86.2	0.288	13.38	0.113	0.16	2.29
S2	4.170 ± 0.480	35.4 ± 5.37	79.89	0.252	12.61	0.048	0.08	0.96
S3	2.970 ± 0.420	2.2 ± 12.6	0.5	0	-	0.071	0.09	0.72
Span 80								
S4	0.070 ± 0.043	−5.98 ± 3.45	53.77	0.17	12.79	0.19	1.2	0.19
S5	0.084 ± 0.062	−2.15 ± 3.70	29.36	0.094	12.78	0.17	1.14	0.17
S6	0.081 ± 0.044	−2.28± 3.61	58.84	0.184	12.48	0.19	1.2	0.19
